# Urinary albumin-to-creatinine ratio and serum albumin are predictors of acute kidney injury in non-ventilated COVID-19 patients: a single-center prospective cohort study

**DOI:** 10.1007/s11255-022-03348-5

**Published:** 2022-09-20

**Authors:** Karolina Schnabel, Nóra Garam, Nóra Ledó, Noémi Hajdú, Ágnes Kóczy, István Takács, Ádám Gy. Tabák, András Tislér

**Affiliations:** 1 Department of Internal Medicine and Oncology, Semmelweis University Faculty of Medicine, Korányi Sándor utca 2/a, Budapest, 1083 Hungary; 2 Department of Public Health, Semmelweis University Faculty of Medicine, Budapest, Hungary; 3 Department of Epidemiology and Health Care, University College London, London, UK

**Keywords:** COVID-19, Acute kidney injury, Clinical nephrology, Fractional excretions

## Abstract

**Purpose:**

Acute kidney injury (AKI) is a frequent complication among COVID-19 patients in the intensive care unit, but it is less frequently investigated in general internal medicine wards. We aimed to examine the incidence, the predictors of AKI, and AKI-associated mortality in a prospective cohort of non-ventilated COVID-19 patients. We aimed to describe the natural history of AKI by describing trajectories of urinary markers of hemodynamic, glomerular, and tubular injury.

**Methods:**

141 COVID-19 patients were enrolled to the study. AKI was defined according to KDIGO guidelines. Urine and renal function parameters were followed twice a week. Multivariate logistic regression was used to determine the predictors of AKI and mortality. Trajectories of urinary markers were described by unadjusted linear mixed models.

**Results:**

19.7% patients developed AKI. According to multiple logistic regression, higher urinary albumin-to-creatinine ratio (OR 1.48, 95% CI 1.04–2.12/1 mg/mmol) and lower serum albumin (OR 0.86, 95% CI 0.77–0.94/1 g/L) were independent predictors of AKI. Mortality was 42.8% in the AKI and 8.8% in the group free from AKI (*p* < 0.0001). According to multiple logistic regression, older age, lower albumin, and AKI (OR 3.9, 95% CI 1.24–12.21) remained independent predictors of mortality. Urinary protein-to-creatinine trajectories were diverging with decreasing values in those without incident AKI.

**Conclusion:**

We found high incidence of AKI and mortality among moderately severe, non-ventilated COVID-19 patients. Its development is predicted by higher albuminuria suggesting that the originally damaged renal structure may be more susceptible for virus-associated effects. No clear relationship was found with a prerenal mechanism, and the higher proteinuria during follow-up may point toward tubular damage.

## Introduction

More than 188 million people were infected with SARS-CoV2 (severe acute respiratory syndrome-related coronavirus 2) worldwide. The clinical characteristic of coronavirus disease (COVID-19) caused by SARS-CoV-2 is highly heterogeneous, ranging from mild flu-like illness to life-threatening multiorgan failure. According to the World Health Organization, over 5 million deaths were caused by COVID-19 [1]. Hungarian data shows that around 120,000 people were affected and 39,000 patients died until January 2022 [2]. In addition to respiratory symptoms, heart, kidney and gastrointestinal involvements are also frequently reported. Based on observational data, both a systemic cytokine storm and endothelial injury could play a role in the pathogenesis of COVID-19-related organ failure including acute kidney injury (AKI) [3, 4].

COVID-19-associated AKI usually develops early in the disease course with hematuria, proteinuria, and with a rapid decline of renal function sometimes necessitating renal replacement therapy [5]. According to a recent meta-analysis, the incidence of AKI in the USA and in Europe was higher (28.6%) compared to China where it was only 5.5% of hospitalized patients. In this study, the pooled incidence was 29% among patients treated in intensive care units [6]. Another study from the USA shows even higher rates of AKI (46%) among hospitalized patients, among whom 19% needed renal replacement therapy. This study found even higher rates of AKI patients (76%), among whom 24% were admitted to intensive care units [6, 7].

Studies utilizing kidney biopsies suggest that both glomerular and tubular injuries are involved in the pathogenesis of COVID-19-associated AKI [8]. Furthermore, the analysis of post-mortem kidney specimens shows that tubular injuries were the predominant lesions, but glomerular damages (such as glomerular collapse and focal segmental sclerosis) were also present. Unfortunately, tubular autolysis could affect post-mortem samples leading to misclassification [8, 9].

Based on the controversy shown above, the pathophysiology of kidney injury associated with COVID-19 is still unclear. According to the most cited hypothesis, the stimulation of ACE-2 receptors in the kidney leads to local release of inflammatory cytokines, which injure the podocytes, as well as endothelial and tubular epithelial cells [10–12]. In addition, systemic effects such as the cytokine storm, interferon suppression through SARS-CoV2 receptors, damaged adaptive immunity, and overactivation of the complement cascade may also contribute to kidney injury [13].

Besides the direct (cytokine related) damage to kidney tissues, prerenal (hemodynamic) mechanisms may also contribute to the pathogenesis of AKI [14]. Prerenal mechanisms (reduced effective circulating volume) can be estimated using fractional excretion of sodium (FeNa), urea (FeUrea), or uric acid (FeUA), which are all decreased if prerenal kidney injury is present [15–17]. While FeNa could be affected by the use of diuretics or the presence of sepsis, FeUrea is much more reliable [18–20]. Furthermore, FeUA is frequently used in the differential diagnosis of hyponatremia [21]. These tests together with the evaluation of urinary protein and albumin excretion may be useful to differentiate between hemodynamic, glomerular, and tubular injuries.

During the past 2 year,s Semmelweis University was one of the centers that provided care for hospitalized COVID-19 patients in Hungary. Our department was nominated for the care of moderately severe, non-ventilated COVID-19 patients admitted to Semmelweis University.

We aimed to determine the (1) incidence and (2) predictors of AKI and (3) the relation between AKI and mortality in a prospective observational study. Our secondary objective was to describe trajectories of FeNa, FeUrea, FeUA, urinary protein-to-creatinine ratio (UPCR) and urinary albumin-to-creatinine ratio (UACR) using twice weekly measurements during hospitalization to gain a better insight into the pathogenesis of COVID-19-associated AKI.

## Materials and methods

### Setting

All adult patients were eligible to participate in the prospective cohort study if they were admitted to the Department of Internal Medicine and Oncology, Semmelweis University, Budapest, Hungary, with a confirmed SARS-CoV-2 infection (based on real-time polymerase chain reaction or direct antigen tests) between 27/NOV/2020 and 15/MAR/2021. Baseline assessment of demography, medical history, laboratory, and imaging assessments, as well as symptomatic and causal treatments were driven by the institutional protocols. In brief, all patients admitted received low-dose low-molecular weight heparin, oral dexamethasone (10 days), azithromycin (3 days), and cholecalciferol (12,000 IU for 5 days, followed by 3000 IU per day). The use of antiviral treatment was based on the degree of pulmonary involvement seen on chest CT scans: no specific antiviral treatment for mild pulmonary involvement, favipiravir treatment for moderate disease (no oxygen requirement) for 7–10 days, remdesivir iv. for patients with more than 25% lung involvement and/or requiring oxygen supplementation for 5–10 days. In addition, reconvalescent plasma therapy, baricitinib, or tocilizumab was used in severe disease on a case-by-case basis.

For participants of this study, we collected urinary samples for the determination of FeNa, FeUrea, FeUA, UPCR, UACR, as well as serum creatinine twice weekly to determine incident AKI and describe hemodynamic, glomerular, and tubular kidney injury.

All study-related procedures were performed in accordance with the ethical standards laid down in the 1964 Declaration of Helsinki and its later amendments. Local ethical approval was obtained from the Semmelweis University Regional and Institutional Committee of Science and Research Ethics (Registration number: SE-RKEB 245–1/2020). All participants signed an informed consent before any study-related procedures were performed.

### Participants

**Fig. 1:**
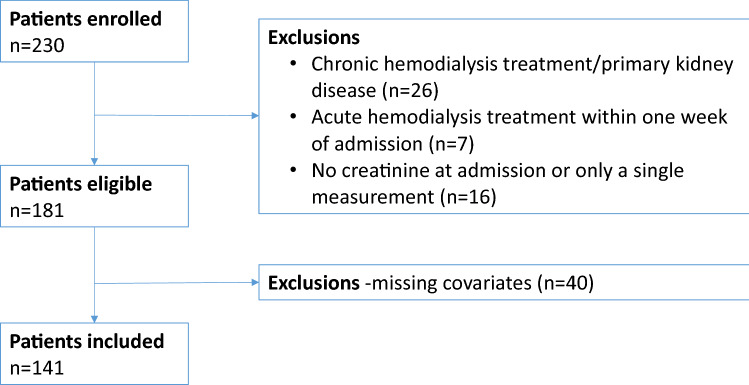
Flowchart of the enrolled patients

Of the potentially eligible patients, *n* = 230 agreed to participate. From these we excluded those with known primary kidney disease, those on regular hemodialysis (*n* = 26), those that required renal replacement therapy within a week of admission (*n* = 7), and those without repeat creatinine determinations (*n* = 16), leaving *n* = 181 patients eligible for inclusion. We further excluded *n* = 40 patients with missing baseline covariates, leading to a final analytical sample of 141 patients (77.9% of those eligible) (Fig. 1).

### Predictors and covariables

On admission, demographic, clinical, and laboratory data were recorded in the electronic health record. Among demographic characteristics, we included patient age and sex.

Of the information on medical history, the presence of hypertension and diabetes was collected (based on the description of the treating physician). All laboratory analyses were performed at the same central laboratory (Department of Laboratory Medicine of Semmelweis University) on automated systems. For the current analysis, we recorded blood cell counts, serum creatinine, urea, sodium, potassium, uric acid, lactate dehydrogenase, procalcitonin, and fibrinogen.

Spot urinary samples as well as sera were collected for the determination of urinary and serum sodium, urea, uric acid, creatinine, as well as urinary albumin and protein for the determination of FeNa, FeUrea, FeUA, UPCR, and UACR twice weekly. Fractional excretion was calculated using the following equation: FeSubstance = (UrineSubstance/SerumSubstance)/(UrineCreatinine/SerumCreatinine) * 100%. As baseline urinary samples were missing for 40 patients, we imputed these data to maximize the sample size for the multivariate analysis based on the estimated individual trajectories of these variables (see details in the Statistical methods).

### Outcomes

*AKI* during follow-up was diagnosed based on the Kidney Disease Improving Global Outcome (KDIGO) criteria with the creatinine value on admission as baseline. Thus, AKI was diagnosed if (1) serum creatinine increased > 26.5 µmol/L within 4 days, or (2) reached a value > 150% of baseline within 7 days. Stage I AKI is diagnosed if the creatinine increase is within 1.5–1.9 times that of baseline, stage II if within 2–2.9 times, and stage III if the creatinine increase is ≥ 3 times that of baseline, or it reaches 354 µmol/L or renal replacement therapy is initiated [22]. For the determination of incident AKI, all serum creatinine measurements (at least twice a week) recorded were used from admission until the end of hospitalization (discharge, transfer to another unit, or death).

In hospital, all-cause mortality was drawn from the electronic health record. Follow-up was censored if the patient was discharged from the university hospital system (to home or an outside institution).

### Statistical analysis

Descriptive data are shown as mean (standard deviation) or median (interquartile range) for continuous variables and *n* (%) for categorical variables as appropriate. All continuous variables were tested for normality by formal normality tests and by visual inspection of the data. Variables with non-normal distributions were log-transformed to improve normality.

Baseline data stratified by incident AKI and survival status were compared using independent sample *t* tests for continuous variables using log-transformed variables as required. Categorical variables were compared by Chi-square tests.

Given the fact that both incident AKI and deceased participants were much older than their respective controls, potential predictors of AKI and mortality were evaluated individually in logistic regression models adjusted for age and sex. Variables that showed an association with the respective outcome (*p* < 0.1) were made available for a multiple logistic regression model of independent predictors where backward stepwise selection was applied.

Based on visual inspection of Loess curves of the individual urinary measures by incident AKI status, a quadratic model seemed to be a good fit for the overall data. We used multilevel longitudinal modeling to trajectories of FeNa, FeUrea, FeUA, UPCR, and UACR during hospitalization [23]. Data were structured so that the repeated measurements (person-observations) of urinary markers were nested within subjects and the non-independence of the person-observations (the same individuals contributed to more than one observation in the dataset) was taken into account in estimating standard errors. Differences in trajectories between AKI cases and controls were modeled using unadjusted linear growth curves with the addition of constant for caseness as well as a time-by-caseness interaction. Estimated marginal means predicted by these linear growth models were back-transformed to the original scale and used for graphical representation of urinary marker trajectories. Furthermore, we saved the model-fitted values for each individual and used the estimated admission value if no baseline measurement was found but at least two measurements were recorded during hospitalization.

Two-tailed *p* values were calculated and significance was established at a *p* value < 0.05. Statistical analysis was done with IBM SPSS Statistics 28.

## Results

### Predictors of AKI

Altogether, 28/141 (19.7%) patients were diagnosed with AKI at a median of 9 (IQR 6–16) days of hospitalization (stage I—*n* = 17, stage II—*n* = 4, stage III—*n* = 7). One patient required hemodialysis treatment during hospitalization. Five patients (2 with AKI and 3 without AKI) were transferred to ICU, among whom four died later in the ICU. Patients with AKI were older, had a substantially higher mortality, higher serum urea, lower serum albumin, higher UPCR and UACR, and a lower FeNa compared to controls (all *p* < 0.05). Procalcitonin tended to be higher among AKI cases (*p* < 0.1). No difference in the sex distribution, frequency of diabetes or hypertension, blood cell count, other measures of kidney function, serum electrolytes, fibrinogen, lactate dehydrogenase, or other urinary markers was found (Table 1).

**Table 1:** Characteristics of COVID-19 patients by incident acute kidney injury status

	Patients with acute kidney injury (*n* = 28)	Patients without acute kidney injury (*n* = 113)	All (*n* = 141)	p*
Age (years)	73.2 (65.8–81.4)	65.4 (52.6–78.1)	67.5 (55.8–78.2)	** < 0.001**
Male (%)	17 (60.7)	60 (53)	77 (54.6)	0.49
Hospitalization (days)	16 (6–21)	9 (6–14)	9 (6–16)	0.12
Mortality (%)	12 (42.8)	10 (8.8)	22 (15.6)	** < 0.001**
Hypertension (%)	20 (71.4)	67 (59.3)	87 (61.7)	0.25
Diabetes mellitus (%)	12 (42.8)	30 (26.5)	42 (29.8)	0.097
Hemoglobin (g/L)	112.6 (21.6)	122.2 (22.1)	120.3 (23.2)	0.41
White blood cell (g/L)	7.64 (4.71–10.09)	6.38 (4.24–8.77)	6.42 (4.32–9.04)	0.24
Platelets (g/L)	208 (136–292)	205 (157–280)	205 (150–288)	0.8
Creatinine (µmol/L)	97 (63–185)	82 (68–109)	84 (66–117)	0.174
Urea (mmol/L)	11 (5.6–16.3)	6.6 (4.8–10.1)	7.1 (4.9–11.3)	**0.012**
Sodium (mmol/L)	139 (135–143)	139 (136–141)	139 (136–142)	0.73
Potassium (mmol/L)	4.4 (3.8–4.6)	4.1 (3.8–4.6)	4.1 (3.8–4.6)	0.71
Uric acid (µmol/L)	345 (254–454)	320 (248–409)	332 (248–417)	0.5
Fibrinogen (g/L)	4.92 (3.92–5.69)	5.16 (4.08–6.08)	5.01 (4–6)	0.15
Albumin (g/L)	28.2 (5.7)	32.9 (4.6)	32 (5.1)	** < 0.001**
Lactate dehydrogenase (U/L)	298 (166–537)	303 (220–424)	302 (215–429)	0.806
Procalcitonin (µg/L)	0.34 (0.09–0.74)	0.11 (0.05–0.30)	0.12 (0.05–0.4)	0.079
Urine protein/creatinine ratio (mg/mmol)	61 (39.9–361.4)	48.6 (34.13–162.4)	50.4(34.5–242.3)	**0.001**
Urine albumin/creatinine ratio (mg/mmol)	9.5 (3.4–126.5)	5.8 (2.7–42.1)	6.1 (2.8–80.6)	**0.005**
FeNa (%)	0.2(0.09–2.23)	0.35 (0.13–2.8)	0.32 (0.13–2.4)	**0.029**
FeUA (%)	5.74 (4.1–17.6)	7.6 (3.7–18.2)	6.8 (3.9–18.2)	0.29
FeUrea (%)	27.5 (17.6–47.9)	32.46 (22.5–55.1)	31.35 (21.1–52.9)	0.58

Two-tailed *p* values were calculated and significance was established at a *p* value < 0.05

Categorical variables defined as *n* (%), continuous variable as median (interquartile range), except for haemoglobin and albumin where mean (standard deviation) is shown

*FeNa* fractional sodium excretion, *FeUA* fractional uric acid excretion, *FeUrea* fractional urea excretion

*p value is a result of the Chi-square and Student’s *t* tests after logarithmic transformation of the data

Given the large age difference, we investigated each potential predictor in individual models adjusted for age and sex. In these models, older age, higher baseline UPCR and UACR, as well as lower serum albumin level were related to the development of AKI (Table 2).

**Table 2:** Individual associations between selected baseline characteristics and incident acute kidney injury based on logistic regression models adjusted for age and sex

	OR	Confidence interval (95%)	*p*
Age (years)	1.048	1.014–1.084	**0.006**
Sex (male)	1.569	0.653–3.771	0.314
Urea (mmol/L)	1.605	0.826–3.119	0.162
UPCR (mg/mmol)	2.2	1.24–3.87	**0.006**
UACR (mg/mmol)	1.51	1.07–2.13	**0.019**
FeNa (%)	1.206	0.89–1.62	0.217
Albumin (g/L)	0.856	0.779–0.94	**0.001**
Procalcitonin (µg/L)	1.115	0.887–1.4	0.35

Two-tailed *p* values were calculated and significance was established at a *p* value < 0.05

*UPCR* urine protein/creatinine ratio, *UACR* urine albumin/creatinine ratio, *OR* odds ratio

In the final multivariable logistic model, only lower baseline albumin and higher UACR remained as independent predictors of AKI (Table 3).

**Table 3:** Independent predictors of acute kidney injury based on multivariate logistic regression models adjusted for age and sex

	OR	Confidence interval (95%)	*p*
Age (years)	1.023	0.98–1.06	0.224
Sex (male)	1.72	0.65–4.53	0.27
Albumin (g/L)	0.856	0.77–0.94	0.002
UACR (mg/mmol)	1.48	1.043–2.12	0.028

Backward stepwise selection of variables

Other variables available for the model: UPCR

*UPCR* urine protein/creatinine ratio, *UACR* urine albumin/creatinine ratio, *OR* odds ratio

### Predictors of mortality

During the median 12-day (IQR 6–16) follow-up, 22 (15.6%) patients died, 12 (42.8%) from the AKI and 10 (8.8%) from the non-AKI group (*p* < 0.001). At the time of admission, deceased patients were older, had lower hemoglobin, higher white blood cell count, urea, and uric acid, lower albumin, higher procalcitonin, and lower UPCR FeNa and FeUrea levels compared to surviving patients (all *p* < 0.05). Although UPCR was overall lower in the deceased group, its range was much wider suggesting that the differences observed here may be less reliable. Deceased patients tended to have lower fibrinogen and UACR levels (*p* < 0.1) (Table 4).

**Table 4:** Characteristics of COVID-19 patients by survival status

	Deceased (*n* = 22)	Alive (*n* = 119)	p*
Age (years)	78.6 (72.8–81.7)	64.5 (52.5–76.5)	** < 0.001**
Male (%)	12 (54.5)	65 (54.6)	0.96
Hospitalization (days)	8.5 (6–18)	9 (6–16)	0.484
Acute kidney injury (%)	12 (54.5)	16 (13.4)	** < 0.001**
Hypertension (%)	15 (68.2)	72 (60.5)	0.525
Diabetes mellitus (%)	6 (27.3)	36 (31)	0.76
Hemoglobin (g/L)	107.3 (23.5)	122.7 (21.2)	**0.002**
White blood cell (g/L)	8.6 (6.4–10.8)	6.3 (4.2–8.7)	**0.007**
Platelets (g/L)	230 (159–328)	205 (147–266)	0.868
Creatinine (µmol/L)	109 (76–194)	81 (66–112)	0.107
Urea (mmol/L)	10.2 (6–13.7)	6.6 (4.8–10.5)	**0.027**
Sodium (mmol/L)	140 (137–143)	139 (136–141)	0.27
Potassium (mmol/L)	4.2 (3.6–4.6)	4.1 (3.8–4.6)	0.73
Uric acid (µmol/L)	408 (284–514)	323 (235–406)	**0.049**
Fibrinogen (g/L)	4.1 (3.6–5.5)	5.23 (4.15–6.1)	0.083
Albumin (g/L)	26.7 (5.9)	32.9 (4.4)	** < 0.001**
Lactate dehydrogenase (U/L)	375 (157–597)	297 (216–418)	0.52
Procalcitonin (µg/L)	0.4 (0.1–0.8)	0.1 (0.05–0.3)	**0.032**
Urine protein/creatinine ratio (mg/mmol)	47.5 (30.8–1032.7)	50.9 (34.5–198.3)	**0.016**
Urine albumin/creatinine ratio (mg/mmol)	5.10 (2.5–483)	6.2 (2.9–49.9)	0.054
FeNa (%)	0.25 (0.07–2.4)	0.3 (0.16–2.8)	**0.01**
FeUA (%)	7.2 (3.5–20.1)	6.75 (4.1–18.2)	** < 0.001**
FeUrea (%)	30.6 (19.7–55.7)	32.5 (21.5–53)	**0.002**

Two-tailed *p* values were calculated and significance was established at a *p* value < 0.05

Categorical variables defined as *n* (%), continuous variable as median (interquartile range), except for haemoglobin and albumin where mean (standard deviation) is shown

*FeNa* fractional sodium excretion, *FeUA* fractional uric acid excretion, *FeUrea* fractional urea excretion

*p value is a result of the Chi-square and Student’s *t* test after logarithmic transformation of the data

In individual multiple logistic regression models adjusted for age and sex, older age (OR 1.08, 95% CI 1.04–1.13/1 year), lower hemoglobin (OR 0.98, 95% CI 0.95–1.00/1 g/L), higher white blood cell (OR 2.82, 95% CI 1.03–7.76/1 g/L), presence of AKI (OR 6.02, 95% CI 2.10–17.30), and lower serum albumin (OR 0.81, 95% CI 0.72–0.90/1 g/L) were related to all-cause in-hospital mortality.

In the multivariable model, only older age, presence AKI, and lower albumin levels remained independent predictors of mortality (Table 5).

**Table 5:** Independent predictors of in-hospital mortality based on multiple logistic regression adjusted for age and sex

	OR	Confidence interval (95%)	*p*
Age (year)	1.068	1.012–1.127	0.017
Sex (male)	0.95	0.31–2.87	0.928
Albumin (g/L)	0.84	0.75–0.94	0.002
Acute kidney injury	3.9	1.24–12.21	0.019

Backward stepwise selection of variables

Other variables available for the model: hemoglobin, white blood cell, UPCR, fibrinogen

*OR* odds ratio

### Trajectories of urinary measures

**Fig. 2:**
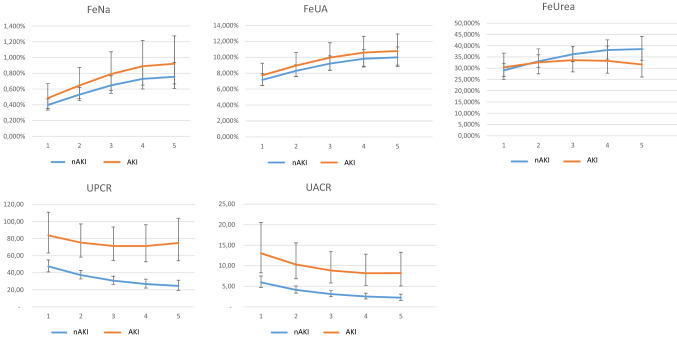
Trajectories of fractional excretion of sodium, uric acid, urea and urinary protein and albumin to creatinine ratio from admission (time point 1) to the end of the second week of hospitalization (time point 5) based on mixed models. Numbers on the x-axis show follow-up points, with two urinary assessments weekly. *AKI* acute kidney injury, *nAKI* patients without acute kidney injury, *FeNa* fractional sodium excretion, *FeUA* fractional uric acid excretion, *FeUrea* fractional urea excretion, *UPCR* urine protein/creatinine ratio, *UACR* urine albumin/creatinine ratio

The trajectories of all urinary markers (raw data or log-transformed as appropriate) followed quadratic trajectories during hospitalization (all *p* < 0.05). For the models of FeNa and FeUA, the caseness-by-time interaction was non-significant and was dropped from the model. The confidence intervals were overlapping for these variables, suggesting no difference in the trajectories of these variables (Fig. 2).

For the fractional excretion of urea, we found a non-significant interaction between caseness and time, but the confidence intervals of the estimated values were completely overlapping for the observation period of five measurements (Fig. 2).

For both UACR and UPCR, we found significantly higher values in the incident AKI group at admission with a significant caseness-by-time interaction for UPCR. Accordingly, the trajectories remained almost parallel for UACR, while for UPCR they were diverging, with a faster decline in controls than in the incident AKI group (Fig. 2).

## Discussion

In this prospective cohort study, we found that AKI was diagnosed in almost a fifth (19.7%) of COVID-19 patients who did not require intensive care. According to a multivariate logistic regression, higher UACR and lower albumin levels were independent predictors of incident AKI. During hospitalization, over 40% of incident AKI patients and only 8.8% of controls died. Furthermore, incident AKI was an independent predictor of mortality in multivariate analysis in addition to older age and lower serum albumin levels. While the trajectories were completely overlapping for FeNa, FeUA, and FeUrea between AKI cases and controls during hospitalization, both urinary protein and albumin excretion were consistently higher in the incident AKI patients compared to controls. However, while these trajectories were parallel for UACR, they were diverging for UPCR.

Literature data clearly indicate that the incidence of AKI is higher among COVID-19 patients compared to those hospitalized for community-acquired pneumonia or sepsis of similar severity [7]. However, the reported incidence rates show high variability: 22–36% in the USA, while only 5–11% in the Republic of China [5, 7, 24]. The potential explanation for these differences could lie in the different characteristics of the studied population (such as disease severity or ethnic composition). The 20% incidence of AKI in our cohort of patients with moderate severity COVID-19 well corresponds to figures in the literature [7, 25]. It should be highlighted that most of our cases represent stage I AKI and the incidence of stage II and III AKI is only 7.8% that is substantially lower than the 15.5% reported in the literature. This further emphasizes that our COVID-19 cases had a ‘mild’ disease course and all patients requiring invasive ventilation or developing multiorgan failure were admitted to the intensive care unit and were mostly excluded from study.

According to reports from all over the globe, acute kidney injury is associated with increased mortality in COVID-19 patients [7, 26]. We found that deceased patients were older, with lower haemoglobin and albumin levels, higher white blood cell counts and procalcitonin levels at baseline, suggesting that patients with wasting disease and those with a potential bacterial superinfection were more vulnerable to a severe course of COVID-19.

Reports from the USA show mortality rates between 34 and 72%, while similar figures from China range from 16.1 to 86.4% [27]. The mortality among our patients with AKI was 42.8% which is in line with previous observations. Furthermore, AKI remained an independent predictor of mortality even after adjustment of age and serum albumin levels. Although the confidence interval is wide, related to the relatively low power of our study, the point estimate of almost four suggests a non-trivial effect size even among COVID-19 patients who did not need intensive care or mechanical ventilation.

The mechanism of the development of AKI among COVID-19 patients is not fully understood and it likely encompasses several mechanisms. The most frequently cited of these are the direct cytotoxicity through ACE-2 receptors, direct tubular injury due to SARS-CoV2 infection, thrombotic events as a result of endothelial damage, complement activation, hypoperfusion as a consequence of hypovolemia or sepsis, and drug toxicity [25, 28–30]. Literature data indicate that patients with AKI are generally older, more frequently male, and have a higher number of comorbidities (such as hypertension, heart failure, known chronic kidney disease) [7, 26]. Among laboratory measurements, higher levels of inflammatory parameters and D-dimer show association with incident AKI. Hematuria and proteinuria were also more frequent in COVID-19-associated kidney disease patients [7, 13, 31–36]. To this end, we compared the characteristics of our patients with and without incident AKI at admission. Similar to the literature, incident AKI patients were older, but we could not find any sex differences stratified by incident AKI. Patients diagnosed later with AKI had a decreased renal function at admission with higher protein excretion than controls. This may suggest that previous, even asymptomatic, and subtle kidney disease is a risk factor for the development of an “acute-on-chronic” worsening of kidney function during COVID-19 infection. In fact, chronic kidney disease is a known risk factor for the development of AKI in other clinical scenarios as well [26, 37]. An alternative explanation for our finding of increased baseline urea levels and proteinuria in incident AKI cases is that these abnormalities are markers of renal damage caused by COVID-19 that had already occurred before admission.

Serum albumin was lower in the AKI group compared to controls and hypoalbuminemia remained an independent predictor of incident AKI. The higher urinary albumin excretion in the AKI group is an unlikely explanation for this finding, as the level of albuminuria was well below of what would lead to hypoalbuminemia. It is rather likely that hypoalbuminemia was a marker of a more severe disease course and poor general condition. Overall, our findings suggest that an originally damaged renal structure may be more susceptible to the virus-associated effects.

Acute kidney injury in COVID-19 can be the result of prerenal/hemodynamic and renal (glomerular, tubular) causes [4, 8, 24, 34]. To entangle the potential role of these mechanisms, we measured fractional excretion of sodium, urea, and uric acid, as well as albuminuria and proteinuria at admission and throughout hospitalization.

Fractional excretion measurements may help identify decreased renal perfusion behind worsening kidney function. AKI of a prerenal cause is mostly associated with FeNa below 1% (although it may be influenced by diuretics or other factors), whereas it is higher in tubular damage [17, 38]. Similarly, FeUrea below 35–40% and FeUA below 10–12% suggest decreased effective circulatory volume [19, 22, 39, 40]. FeNa, FeUA, as well as FeUrea were low on admission in our study and showed increasing trajectories during the hospital stay with no difference between incident AKI cases and controls. The uniformly low admission value of these markers suggests decreased effective circulatory volume on admission, likely due to infection, fever, dehydration, etc. The increasing trajectory during follow-up probably reflects the effect of overall medical care such as fluid management and avoidance of low blood pressure. The fact that incident AKI and control patients had overlapping trajectories makes “prerenal” mechanisms an unlikely cause of COVID-19-related incident AKI in our cohort. To the best of our knowledge, fractional excretion among COVID-19 patients was studied only by one other research group. They reported higher FeNa values among patients who later developed AKI [32].

Elevated urinary protein levels are associated with both glomerular and tubular injuries. According to the literature, elevated urinary protein levels in AKI associated with COVID-19 are mostly a result of the presence of low molecular weight proteins (not albumin) that could be a sign of decreased reabsorption due to injured tubular function [23]. In COVID-19, damage of the proximal tubule could lead to low molecular weight protein excretion, aminoaciduria, or partial Fanconi syndrome as well [7]. In our study, both urinary albumin and urinary protein excretion were higher in the incident AKI group compared to controls at admission. Urinary albumin excretion decreased in both groups during follow-up. In contrast, total protein excretion decreased only in controls and remained elevated in those with incident AKI. This observation suggests the hypothesis that elevated protein excretion is not a consequence of albuminuria, and that it may be low molecular weight proteinuria due to tubular damage. The different patterns of trajectories of albuminuria and proteinuria in our patients suggest that the tubular component may be the driver of the development of AKI in COVID-19 patients.

The limitations of our study need to be acknowledged. Our cohort includes patients with moderate-severity COVID-19 that limits the generalizability to more severe COVID-19 cases. While we made efforts to maximize data collection, the number of included AKI cases was limited that leads to wide confidence intervals. On the one hand, we cannot exclude the possibility that we missed some important associations; on the other, the strengths of the observed associations cannot be precisely estimated. As this study was conducted in a non-ICU COVID medical ward, hourly or 6-hourly urine collections (as required by the definition of KDIGO for AKI) were not performed. Therefore, we used only the criteria based on the most reliable marker, the creatinine value to diagnose AKI. While omitting urine output measurement likely decreased the incidence of AKI, the use of the creatinine-based diagnosis provides an underestimation of the high incidence of AKI in this clinical setting. Similarly, we used a spot urine to determine urine protein-to-creatinine, urine albumin-to-creatinine ratios, and fractional excretion indexes instead of the “gold standard” of a 24-h timed urine collection. However, literature data suggest that UPCR and UACR are reliable estimates of daily excretion values [41]. Furthermore, we had to exclude over 20% of potential participants due to missing data that could introduce selection and other causes of bias into our analysis. Due to the low number of more severe AKI cases, we could not further stratify AKI cases to better examine the suspected prerenal and tubular damages. Unfortunately, we could not avoid the effect of diuretics or steroids on fractionated excretion. While this could have had a large effect on FeNa values, the other fractional excretion markers are less prone to the effect of different medications [19]. Overall, the fact that all fractional excretion markers point to the same direction strengthen our conclusion. Finally, we did not perform kidney biopsies to get insight into the renal structural changes.

At the same time, the strength of the study is that our observation concentrates on a less studied population with moderate disease and our cohort was well phenotyped. The most important and unique strength of our study is the ability to describe the natural history of COVID-19-related renal changes by the evaluation of temporal changes of urinary parameters over the course of hospitalization.

In conclusion, the incidence and mortality of AKI are relatively high among hospitalized, moderately severe, non-ventilated COVID-19 patients. It is plausible that an already impaired renal function, as suggested by higher baseline proteinuria, makes the kidneys more susceptible to virus-associated effects. While no clear relationship was found with prerenal markers, the increased proteinuria in incident AKI at baseline and the diverging trajectories between incident AKI cases and controls with COVID-19 during follow-up may point to the possibility of tubular damage in the development of AKI associated with COVID-19. This hypothesis, however, needs further confirmation in independent studies.
